# Design, Synthesis, and Biological Evaluation of Novel 6*H*-Benzo[*c*]chromen-6-one Derivatives as Potential Phosphodiesterase II Inhibitors

**DOI:** 10.3390/ijms22115680

**Published:** 2021-05-26

**Authors:** Long Tang, Jianchun Jiang, Guoqiang Song, Yajing Wang, Ziheng Zhuang, Ying Tan, Yan Xia, Xianfeng Huang, Xiaoqing Feng

**Affiliations:** 1 Institute of Chemical Industry of Forest Products, Chinese Academy of Forestry, Nanjing 210042, China; 00009205@cczu.edu.cn; 2 School of Chemical Engineering, Nanjing Forestry University, Nanjing 210037, China; 3 School of Pharmaceutical Engineering & Life Science, Changzhou University, Changzhou 213164, China; sgq@cczu.edu.cn (G.S.); cathywang@cczu.edu.cn (Y.W.); zzh2010@cczu.edu.cn (Z.Z.); 15435108@smail.cczu.edu.cn (Y.T.); xy19105500550xy@163.com (Y.X.); huangxf@cczu.edu.cn (X.H.)

**Keywords:** alkoxylated 6*H*-benzo[*c*]chromen-6-one, phosphodiesterase II, inhibitors, biological activities

## Abstract

Urolithins (hydroxylated 6*H*-benzo[*c*]chromen-6-ones) are the main bioavailable metabolites of ellagic acid (EA), which was shown to be a cognitive enhancer in the treatment of neurodegenerative diseases. As part of this research, a series of alkoxylated 6*H*-benzo[*c*]chromen-6-one derivatives were designed and synthesized. Furthermore, their biological activities were evaluated as potential PDE2 inhibitors, and the alkoxylated 6*H*-benzo[*c*]chromen-6-one derivative **1f** was found to have the optimal inhibitory potential (IC_50_: 3.67 ± 0.47 μM). It also exhibited comparable activity in comparison to that of BAY 60-7550 in vitro cell level studies.

## 1. Introduction

Ellagic acid (EA) is an important part of the pharmacological activity of mellow compounds, which are widely distributed in the bark, root, leaf, and fruit of plants. It has a wide range of pharmacological activities, such as antioxidation, antitumor, antimutation, antibacterial, anti-inflammatory, antiviral, and myocardial protection [1]. In recent years, EA showed great therapeutic potential in the treatment of neurodegenerative diseases such as typical Alzheimer’s disease (AD) and Parkinson’s disease (PD) because of its neuroprotective effects and ability to delay neurodegeneration of aging in the mouse model of AD [2,3,4,5,6,7,8]. However, the applications of EA were limited in the field of health care products and cosmetics due to poor oral bioavailability. There are no EA derivatives available on the market in the pharmaceutical field.

Urolithins are main intestinal metabolites, which are derived from the metabolism of EA through tannolactone ring and dihydroxylation [9,10,11,12]. Urolithins have more advantages in absorption than EA, which may be the final material basis for the biological activities of EA [13]. Urolithins are widely distributed in the urine, feces, and bile of mammals such as humans, rats, mice, cattle, and pigs [13,14,15]. The reports on the biological activity of urolithins mainly focus on the antioxidation, anti-inflammatory, and antitumor properties, as well as its regulation of intestinal flora and inhibition of end products of protein glycosylation [9,10,13,16,17]. Some studies reported that both Urolithin A and Urolithin B are involved in neuroprotection because they can significantly inhibit the formation of protein glycosylation end products [18]. Urolithin A can activate neuroprotective mechanisms, such as activating SIRT-1 in differentiated ReNcell VM human nerve cells. SIRT-1 is a deacetylase that regulates DNA expression, cell apoptosis, and aging, and participates in the physiological or pathological processes of organisms by deacetylating substrate proteins. The activation of SIRT-1 is related to the neuroprotective effect of Urolithin A in nerve cells [19]. Urolithin B can cross the blood-brain barrier and has few side effects on the body, so it may provide therapeutic benefits for neurodegenerative diseases associated with oxidative stress and microglial activation [20]. Therefore, urolithins show great therapeutic potential in the treatment of neurodegenerative diseases.

Phosphodiesterase inhibition received much attention for the potential treatment of central nervous system (CNS) disorders. The role of PDEs in the augmentation of cyclic nucleotide signaling makes these enzymes as attractive targets for the enhancing neuronal communication. PDE2A is the only subtype of PDE2 in mammalian species, which is mainly expressed in brain and heart, but remains low in peripheral tissues [21,22,23,24]. Compared with normal people, the level of PDE2A in the brain of AD patients is increased. Laurent Gomez et al. summarized the development of PDE2 inhibitors for the treatment of cognitive disorders [25,26]. In 2013, Zhu et al. reported the composite crystal structure of PDE2 and the highly selective inhibitor BAY 60-7550 (protein number: 4HTX, http://www.rcsb.org, accessed on 20 March 2021), which provided the basis for the rational design of small molecule ligands targeted to PDE2 [27]. However, there are no PDE2 drugs in the markets so far. Therefore, further studies are urgently needed to develop PDE2 inhibitors to meet clinical needs.

In the present study, urolithins were selected as leading compounds, and a series of novel urolithin(6*H*-benzo[*c*]chromen-6-one)-derivatives were designed and synthesized to screen PDE2 inhibitors. The potential neuroprotective effects of resultant compounds were evaluated through PDE2 inhibition effects firstly. The compounds with higher PDE2 inhibition activity were further evaluated by corticosterone-induced cytotoxicity in the hippocampal HT-22 cells.

## 2. Results and Discussion

### 2.1. Synthetic Pathways

**Scheme 1:**
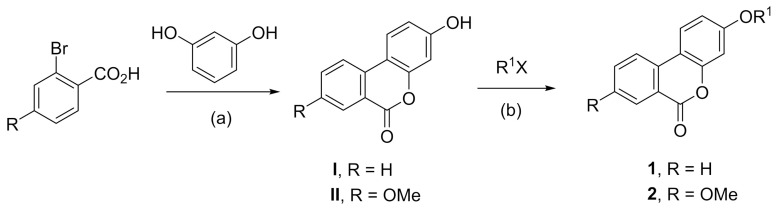
Synthesis of 6*H*-benzo[*c*]chromen-6-one derivatives 1 and 2. Reagents and conditions: (**a**) 5% CuSO_4_, NaOH, H_2_O, reflux, 1.2 h; (**b**) K_2_CO_3_, DMF, 80~120 °C, 3.5~30 h.

Our initial exploration focused on introducing different substituents into the hydroxy scaffold at the 3-position of urolithin while keeping the lactone ring fixed. Thus, various kinds of novel 6*H*-benzo[*c*]chromen-6-one derivatives were designed and synthesized. The general routes for synthesizing 6*H*-benzo[*c*]chromen-6-one derivatives 1 and 2 are shown in Scheme 1 [28]. First, the reaction of 2-bromobenzoic acids and resorcinol in the presence of CuSO_4_ and NaOH provides the intermediate I and II. Next, intermediate I and II were reacted with various kinds of halides including iodomethane, alkyl bromides, and heterocyclic bromides to generate the desired 6*H*-benzo[*c*]chromen-6-one derivatives 1 and 2 in moderate to good yields.

### 2.2. Enzymatic Assays

**Figure 1:**
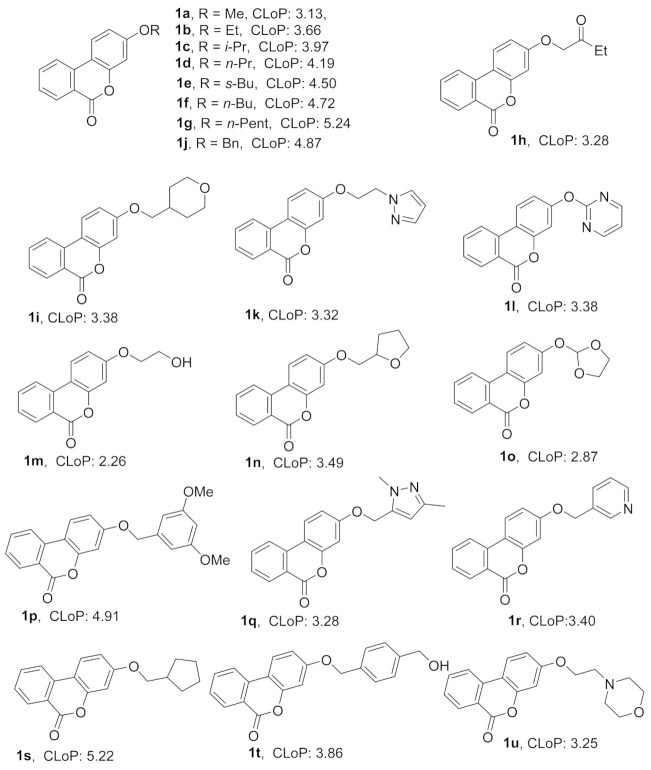
CLoP values of 6*H*-benzo[*c*]chromen-6-one derivatives 1.

**Figure 2:**
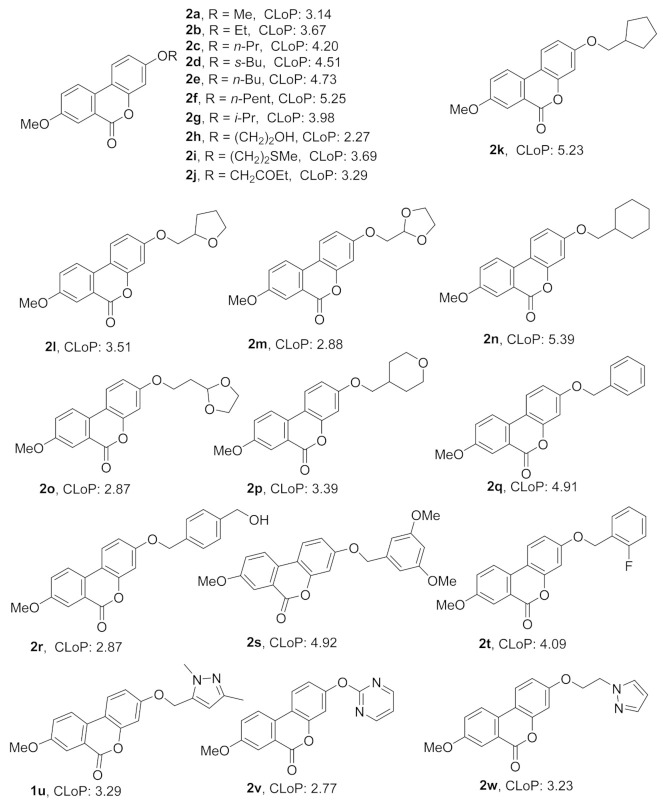
CLoP values of 6*H*-benzo[*c*]chromen-6-one derivatives 2.

Our previous work reported [29] that a propylphenyl group in the classic PDE2 inhibitor BAY 60-7550 was well-accommodated through a hydrophobic-induced binding pocket located under L770 of the protein. Therefore, for urolithin-derivatives in this work, only by bearing certain length and lipophilicity can the R^1^ group interact properly within the pocket. Based on this rule, the 6*H*-benzo[*c*]chromen-6-one derivatives 1 and 2 were evaluated based on their effects on PDE2 inhibitory activities. As shown in Figure 1 and Figure 2, most of the derivatives gave the appropriate values of ClogP (2.0–5.0), indicating the good blood-brain barrier penetration. Moreover, compounds 1a–u and 2a–w were also tested in vitro for PDE2 inhibitory activities, showing that R^1^ group remarkably affected the PDE2 inhibitory activities (as illustrated in Table 1). For compounds 1a–u, when R^1^ group alkane substituted groups with less than five carbons, the PDE2 inhibitory activity is relatively good. To our delight, compound 1**f** significantly affected the PDE2 inhibitory activities (IC_50_ = 3.67 ± 0.47 μM). For compounds 2**a**–**w**, although compound 2**i** had the best PDE2 inhibitory activity, it also needed to be reformed.

### 2.3. Cell Assays

Corticosterone can induce PDE2A, PDE3B, PDE4A, PDE4D, PDE10, and PDE11 expression in HT-22 cells, which results in significant cell lesion [30,31]. Thus, corticosterone-induced HT-22 neurotoxicity was generally employed as classical cell model to screen PDE-inhibitors for curing neurodegenerative disease, such as AD and PD.

**Figure 3:**
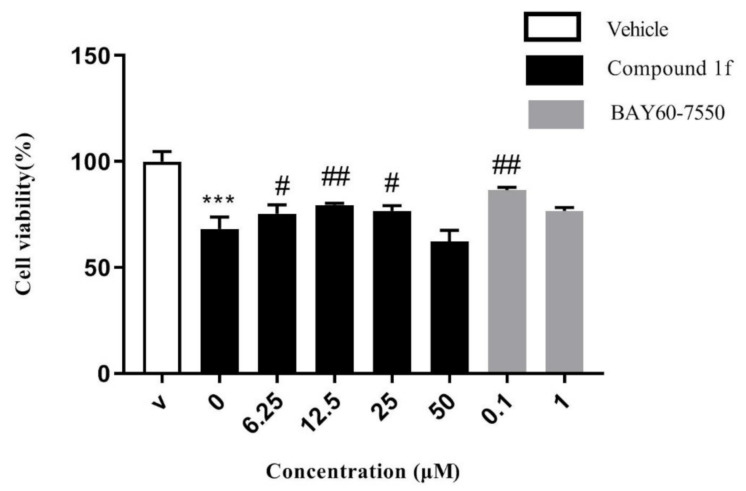
Compound 1f protected cells against corticosterone (CORT)-induced cytotoxicity in a concentration-dependent manner. HT-22 cells were treated with 100 M CORT for 30 min and compound 1f was added for 24 h. Cell viability was measured by MTS assay. Results are expressed as the mean ± standard error of the mean (SEM) of six independent experiments performed in triplicates. *** *p* < 0.001, compared to control group; # *p* < 0.05 and ## *p* < 0.01, compared to vehicle-treated CORT group.

To determine whether compound 1**f** could promote neuron proliferation and protect HT-22 cells against corticosterone-induced neurotoxicity by PDE2 inhibition, the viability of HT-22 cells was measured in the presence of 100 μM corticosterone (positive control), corticosterone absence (control), and different concentrations of 1f. As shown in Figure 3, the cell viability was significantly decreased when HT-22 cells were exposed to 100 μM corticosterone (*p* < 0.001), which indicated that HT-22 neurotoxicity model was successfully built. Compound 1**f** significantly increased cell viability of HT-22 cells in a dose-dependent manner at concentration of 6.25~25 μM, and the best concentration was 12.5 μM (*p* < 0.01). In addition, the effect was similar with the BAY 60-7550 at a concentration of 1.0 μM. Our results showed compound 1**f** possessed good protective effects on HT-22 cells, which was associated with PDE2 inhibition.

## 3. Experiments

### 3.1. Synthesis

All chemicals, reagents, and solvents were analytical grade, purchased from commercial suppliers (i.e., Aladdin, Adamas, and Acros).

The reactions were monitored via thin-layer chromatography (TLC) performed on HSGF254 plates and visualized under UV light. Melting points were measured with an X4-A microscopic melting point apparatus. ^1^HNMR spectra were recorded on a BrukerBioSpin GmbH spectrometer at 300 and 400 MHz; ^13^CNMR spectra were recorded on a BrukerBioSpin GmbH spectrometer at 75 and 100 MHz; the coupling constants are given in Hz. Mass (HRMS) analysis was obtained using Agilent 6200 Accurate-Mass TOF LC/MS system with Electrospray Ionization (ESI). HPLC analysis was obtained using SHIMADZULC-20AB.

#### 3.1.1. 3-Hydroxy-6*H*-benzo[*c*]chromen-6-one (I)

2-Bromobenzoic acid (30 mmol, 6.03 g), resorcinol (60 mmol, 6.60 g), sodium hydroxide (60 mmol, 0.8 g), and water (30 mL) were added in the 100 mL sealed tub, and the reaction was stirred 20 min at 100 °C. Then, 5% copper sulfate solution (9.6 mL) was added to the system and heated to reflux for 1 h. The progress of reaction was monitored through TLC (PE:EA = 5:1). At the end of the reaction, the reaction solution was added dropwise to the ice-water mixture, and the filtered solid continued to be washed with water. The filter cake was recrystallized twice with methanol and glacial acetic acid to obtain compound I (3.50 g, 55.0%) [26]. M.p. 250–251 °C. ^1^H NMR (300 MHz, DMSO-*d*_6_) *δ* = 10.36 (s, 1H), 8.27 (dt, *J =* 8.0, 3.0 Hz, 1H), 8.21–8.15 (m, 2H), 7.89 (m, 1H), 7.57 (m, 1H), 6.85 (dd, *J =* 9.0, 3.0 Hz, 1H), 6.76 (d, *J =* 3.0 Hz, 1H); ^13^C NMR (75 MHz, DMSO-*d*_6_) *δ* = 161.06, 160.32, 152.57, 135.65, 135.52, 130.11, 128.04, 125.21, 122.02, 119.39, 113.60, 109.82, 103.40; HRMS (ESI) *m*/*z* calcd for C_13_H_9_O_3_^+^[M + H]^+^: 213.0473; found: 213.0470. Chromatographic purity: 98.0% (HPLC).

#### 3.1.2. General Procedures for Synthesis of Compounds 1**a**–**u** [28]

The anhydrous DMF (30 mL) was added to a 250 mL round bottom flask, compound I (9.4 mmol, 2.0 g), anhydrous K_2_CO_3_ (12.2 mmol, 1.7 g), and halide (12.2 mmol) were added, and the temperature was controlled at 80~120 °C; the reaction was stirred for 24 h. The reaction was monitored by TLC (PE:EA = 3:1). When the reaction was over, the reaction solution was added to the ice-water mixture to obtain brown crude solid. After suction filtration and drying, the brown crude solid was purified by column chromatography to obtain compounds 1**a**–**u**.

3-Methoxy-6H-benzo[c]chromen-6-one (**1a**): Using iodomethane as the starting material, the desired white solid **1a** was isolated (1.30 g, 61%). M.p. 131.5–132.8 °C. ^1^H NMR (400 MHz, DMSO-*d*_6_) *δ* = 8.35 (d, *J =* 8.0 Hz, 1H), 8.29 (d, *J =* 12.0 Hz, 1H), 8.22 (dd, *J =* 8.0 Hz, 1H), 7.95–7.90 (m, 1H), 7.62 (t, *J =* 6.0 Hz, 1H), 7.06–6.99 (m, 2H), 3.88 (s, 3H); ^13^C NMR (100 MHz, DMSO-*d*_6_) *δ* = 161.67, 160.96, 152.54, 135.78, 135.19, 130.14, 128.53, 125.17, 122.40, 119.68, 112.78, 111.09, 101.91, 56.23; HRMS (ESI) *m*/*z* calcd for C_14_H_11_O_3_^+^[M + H]^+^: 227.0630; found: 227.0626. Chromatographic purity: 98.2% (HPLC).

Ethoxy-6H-benzo[c]chromen-6-one (**1b**): Using bromoethane as the starting material, the desired yellow solid **1b** was isolated (1.70 g, 75%). M.p. 115.2–117.7 °C. ^1^H NMR (300 MHz, DMSO-*d*_6_) *δ* = 8.35–8.22 (m, 3H), 7.93 (t, *J* = 6.0 Hz, 1H), 7.62 (t, *J* = 6.0 Hz, 1H), 6.98 (d, *J* = 6.0 Hz, 2H), 4.13 (q, *J* = 3.0 Hz, 2H), 1.39 (t, *J* = 6.0 Hz, 3H); ^13^C NMR (75 MHz, DMSO-*d*_6_) *δ* = 160.97, 152.55, 135.81, 135.25, 130.15, 128.52, 125.20, 122.41, 119.68, 113.10, 110.99, 102.31, 64.28, 14.94; HRMS (ESI) *m*/*z* calcd for C_15_H_13_O_3_^+^[M + H]^+^: 241.0786; found: 241.0782. Chromatographic purity: 97.6% (HPLC).

3-Isopropoxy-6H-benzo[c]chromen-6-one (**1c**): Using 2-bromopropane as the starting material, the desired yellow solid **1c** was isolated (1.70 g, 71%). M.p. 58.7–59.1 °C. ^1^H NMR (300 MHz, DMSO-*d*_6_) *δ* = 8.68–7.33 (m, 5H), 6.96 (s, 2H), 4.77 (m, 1H), 1.32 (d, *J =* 6.0 Hz, 6H); ^13^C NMR (75 MHz, DMSO-*d*_6_) *δ* = 160.98, 159.92, 152.59, 135.76, 135.23, 130.13, 128.45, 125.22, 122.34, 119.65, 113.83, 110.83, 103.23, 70.42, 22.13; HRMS (ESI) *m*/*z* calcd for C_16_H_15_O_3_^+^[M + H]^+^: 255.0943; found: 255.0940. Chromatographic purity: 97.8% (HPLC).

3-Propoxy-6H-benzo[c]chromen-6-one (**1d**): Using 1-bromopropane as the starting material, the desired white solid **1d** was isolated (1.99 g, 83%). M.p. 67.6–69.2 °C. ^1^H NMR (300 MHz, DMSO-*d*_6_) *δ* = 8.28 (t, *J* = 6.0 Hz, 1H), 8.23–8.17 (m, 2H), 7.92–7.86 (m, 1H), 7.61–7.55 (m, 1H), 6.99–6.93 (m, 2H), 4.04–3.99 (m,2H), 1.83–1.71 (m, 2H), 1.01 (t, *J* = 6.0 Hz, 3H); ^13^C NMR (75 MHz, DMSO-*d*_6_) *δ* = 161.07, 160.95, 152.50, 135.74, 135.21, 130.11, 128.46,125.12, 122.35,119.64, 113.06, 110.95,102.29, 70.00, 22.35, 10.80; HRMS (ESI) *m*/*z* calcd for C_16_H_15_O_3_^+^[M + H]^+^: 255.0943; found: 255.0940. Chromatographic purity: 98.9% (HPLC).

3-(Sec-butoxy)-6H-benzo[c]chromen-6-one (**1e**): Using 2-bromobutane as the starting material, the desired yellow solid **1e** was isolated (1.79 g, 71%). M.p. 73.2–75.8 °C. ^1^H NMR (400 MHz, DMSO-*d*_6_) *δ* = 8.36–8.19 (m, 3H), 7.93–7.88 (m, 1H), 7.62–7.57 (m, 1H), 7.00–6.97 (m, 2H), 4.60–4.53 (m, 1H), 1.74–1.58 (m, 2H), 1.28 (d, *J* = 8.0 Hz, 3H), 0.95 (t, *J* = 4.0 Hz, 3H); ^13^C NMR (100 MHz, DMSO-*d*_6_) *δ* = 160.97, 160.25, 152.58, 135.73, 135.24, 130.12, 128.41, 125.19, 122.31, 119.64, 113.83, 110.83, 103.21, 75.23, 28.89, 19.35, 9.92; HRMS (ESI) *m*/*z* calcd for C_17_H_17_O_3_^+^[M + H]^+^: 269.1099; found: 269.1095. Chromatographic purity: 99.2% (HPLC).

3-butoxy-6H-benzo[c]chromen-6-one (**1f**): Using 1-bromobutane as the starting material, the desired yellow solid **1f** was isolated (1.41 g, 56%). M.p. 53.6–55.5 °C. ^1^H NMR (300 MHz, DMSO-*d*_6_) *δ* = 8.34–8.20 (m, 3H), 7.91 (t, *J* = 9.0 Hz, 1H), 7.60 (t, *J* = 9.0 Hz, 1H), 7.01–6.99 (d, *J* = 6.0 Hz, 2H), 4.09 (t, *J* = 6.0 Hz, 2H), 1.74(t, *J* = 9.0 Hz, 2H), 1.50–1.42 (m, 2H), 0.95 (t, *J* = 9.0 Hz, 3H); ^13^C NMR (75 MHz, DMSO-*d*_6_) *δ* = 161.13, 160.98, 152.54, 135.78, 135.25, 130.14, 128.49, 125.16, 122.39, 119.67, 113.13, 110.98, 102.34, 68.30, 31.03, 19.17, 14.15; HRMS (ESI) *m*/*z* calcd for C_17_H_17_O_3_^+^[M + H]^+^: 269.1099; found: 269.1095. Chromatographic purity: 98.7% (HPLC).

3-(pentyloxy)-6H-benzo[c]chromen-6-one (**1g**): Using 1-bromopentane as the starting material, the desired yellow solid **1g** was isolated (1.49 g, 56%). M.p. 69.4–71.8 °C. ^1^H NMR (400 MHz, DMSO-*d*_6_) *δ* = 8.34–8.20 (m, 3H), 7.91 (t, *J* = 8.0 Hz, 1H), 7.60 (t, *J* = 8.0 Hz, 1H), 7.01–6.98 (m, 2H), 4.08 (t, *J* = 6.0 Hz, 2H), 1.79–1.72 (m, 2H), 1.46–1.32 (m, 4H), 0.91 (t, *J* = 8.0 Hz, 3H); ^13^C NMR (100 MHz, DMSO-*d*_6_) *δ* = 161.10, 160.97, 153.65, 135.77, 135.23, 130.13, 128.47, 125.14, 122.37, 119.66, 113.10, 110.95, 103.11, 68.56, 29.42, 28.12, 22.36, 17.18; HRMS (ESI) *m*/*z* calcd for C_18_H_19_O_3_^+^[M + H]^+^: 283.1256; found: 283.1252. Chromatographic purity: 99.2% (HPLC).

ethyl 2-((6-oxo-6H-benzo[c]chromen-3-yl)oxy)acetate (**1h**): Using ethyl 2-bromoacetate as the starting material, the desired yellow solid **1h** was isolated (1.40 g, 50%). M.p. 124.7–125.8 °C. ^1^H NMR (300 MHz, DMSO-*d*_6_) *δ* = 8.36–8.21 (m, 3H), 7.93 (t, *J* = 7.5 Hz, 1H), 7.62 (t, *J* = 7.5 Hz, 1H), 7.04 (dt, *J* = 9.0, 3.0 Hz, 2H), 4.94 (s, 2H), 4.20 (q, *J* = 7.0 Hz, 2H), 1.24 (t, *J* = 7.5Hz, 3H); ^13^C NMR (75 MHz, DMSO-*d*_6_) *δ* = 168.79, 160.88, 159.94, 152.37, 136.32, 135.03, 130.17, 128.74, 125.29, 122.52, 120.20, 113.06, 111.79, 103.34, 66.14, 62.31, 15.96; HRMS (ESI) *m*/*z* calcd for C_17_H_15_O_5_^+^[M + H]^+^: 299.0841; found: 299.0837. Chromatographic purity: 98.3% (HPLC).

3-((tetrahydro-2H-pyran-4-yl)methoxy)-6H-benzo[c]chromen-6-one (**1i**): Using 4-(bromomethyl)tetrahydro-2H-pyran as the starting material, the desired yellow solid **1i** was isolated (1.60 g, 55%). M.p. 133.0–134.8 °C. ^1^H NMR (300 MHz, CDCl_3_) *δ* = 8.34 (d, *J* = 9.0 Hz, 1H), 7.96 (dd, *J* = 18.0, 9.0 Hz, 2H), 7.78 (t, *J* = 7.5 Hz, 1H), 7.50 (t, *J* = 7.5 Hz, 1H), 6.91–6.82 (m, 2H), 4.04 (dd, *J* = 12.0, 9.0 Hz, 2H), 3.87 (t, *J* = 6.0Hz, 2H), 3.46 (t, *J* = 12.0 Hz, 2H), 2.17–2.06 (m, 1H), 1.80 (d, *J* = 3.0 Hz, 2H), 1.57–4.13 (m, 2H); ^13^C NMR (75 MHz, DMSO-*d*_6_) *δ* = 161.09, 160.97, 152.53, 135.79, 135.22, 130.15, 128.53, 125.19, 122.41, 119.69, 113.13, 111.08, 102.44, 73.00, 67.06, 34.77, 29.60; HRMS (ESI) *m*/*z* calcd for C_19_H_19_O_4_^+^[M + H]^+^: 311.1205; found: 311.1201. Chromatographic purity: 97.3% (HPLC).

3-(Benzyloxy)-6H-benzo[c]chromen-6-one (**1j**): Using (bromomethyl)benzene as the starting material, the desired yellow solid 1j was isolated (2.81 g, 99%). M.p. 113.2–115.3 °C. ^1^H NMR (300 MHz, DMSO*-d_6_*) *δ* = 8.31 (dd, *J* = 15.0, 9.0 Hz, 2H), 8.21 (dd, *J* = 9.0, 1.2 Hz, 1H), 7.94–7.89 (m, 1H), 7.61–7.58 (m, 1H), 7.52–7.48 (m, 2H), 7.45–7.35 (m, 3H), 7.12–7.07 (m, 2H), 5.24 (s, 2H); ^13^C NMR (75 MHz, DMSO*-d_6_*) *δ* = 160.95, 160.70, 152.48, 136.92, 135.81, 135.17, 130.16, 128.99, 128.61, 128.53, 128.37, 125.26, 122.47, 119.76, 113.44, 111.34, 102.91, 70.25; HRMS (ESI) *m*/*z* calcd for C_20_H_15_O_3_^+^ [M + H]^+^: 303.0943; found: 303.0940. Chromatographic purity: 96.8% (HPLC).

3-(2-(1H-pyrazol-1-yl)ethoxy)-6H-benzo[c]chromen-6-one (**1k**): Using 1-(2-bromoethyl)-1H-pyrazole as the starting material, the desired white solid **1k** was isolated (1.24 g, 43%). M.p. 164.3–166.6 °C. ^1^H NMR (300 MHz, DMSO-*d*_6_) *δ* = 8.32 (d, *J* = 9.0 Hz, 1H), 8.27–8.19 (m, 2H), 7.93–7.88 (m, 1H), 7.82 (d, *J* = 2.1 Hz, 1H), 7.63–7.57 (m, 1H), 7.48 (d, *J* = 3.0 Hz, 1H), 7.01–6.95 (m, 2H), 6.27 (t, *J* = 3.0 Hz, 1H), 4.55 (m, 2H), 4.46 (m, 2H); ^13^C NMR (75 MHz, DMSO-*d*_6_) *δ* = 160.94, 160.43, 152.48, 139.39, 135.84, 135.12, 131.08, 130.17, 128.67, 125.29, 122.49, 119.77, 113.16, 111.46, 105.72, 102.63, 67.60, 50.93; HRMS (ESI) *m*/*z* calcd for C_18_H_15_N_2_O_3_^+^[M + H]^+^: 307.1004; found: 307.1001. Chromatographic purity: 96.5% (HPLC).

3-(Pyrimidin-2-yloxy)-6H-benzo[c]chromen-6-one (**1l**): Using 2-bromopyrimidine as the starting material, the desired yellow solid **1l** was isolated (1.83 g, 67%). M.p. 174.0–176.6 °C. ^1^H NMR (400 MHz, DMSO-*d*_6_) *δ* = 8.70 (d, *J* = 8.0 Hz, 2H), 8.45(d, *J* = 8.0 Hz, 2H), 8.28 (d, *J* = 12.0 Hz, 1H), 7.98 (t, *J* = 10.0 Hz, 1H), 7.70 (t, *J* = 10.0 Hz, 1H), 7.41–7.29 (m, 3H); ^13^C NMR (100 MHz, DMSO-*d*_6_) *δ* = 164.80, 160.66, 154.69, 151.92, 135.92, 134.55, 130.26, 129.58, 125.31, 123.09, 120.57, 119.02, 117.93, 115.50, 111.00; HRMS (ESI) *m*/*z* calcd for C_17_H_11_N_2_O_3_^+^[M + H]^+^: 291.0691; found: 291.0686. Chromatographic purity: 98.2% (HPLC).

3-(2-Hydroxyethoxy)-6H-benzo[c]chromen-6-one (**1m**): Using 2-bromoethanol as the starting material, the desired white solid **1m** was isolated (2.34 g, 98%). M.p. 125.7–126.4 °C. ^1^H NMR (400 MHz, DMSO-*d*_6_) *δ* = 8.32 (d, *J* = 8.0 Hz, 1H), 8.27–8.24 (m, 1H), 8.21 (dd, *J* = 8.0, 4.0 Hz, 1H), 7.94–7.90 (m, 1H), 7.61 (t, *J* = 8.0 Hz, 1H), 7.03–7.00 (m, 2H), 5.01 (t, *J* = 6.0 Hz, 1H), 4.11 (t, *J* = 4.0 Hz, 2H), 3.76 (q, *J* = 4.0 Hz, 2H); ^13^C NMR (100 MHz, DMSO-*d*_6_) *δ* = 161.16, 161.07, 152.52, 135.90, 135.23, 130.19, 128.61, 125.23, 122.42, 119.67, 113.25, 111.05, 102.43, 70.65, 59.87; HRMS (ESI) *m*/*z* calcd for C_15_H_13_O_4_^+^[M + H]^+^: 257.0736; found: 257.0732. Chromatographic purity: 98.3% (HPLC).

3-((Tetrahydrofuran-2-yl)methoxy)-6H-benzo[c]chromen-6-one (**1n**): Using 2-(bromomethyl)tetrahydrofuran as the starting material, the desired yellow solid **1n** was isolated (1.31 g, 47%). M.p. 82.8–84.1 °C. ^1^H NMR (400 MHz, CDCl_3_) *δ* = 8.36 (d, *J* = 8.0 Hz, 1H), 8.00 (d, *J* = 8.0 Hz, 1H), 7.94 (d, *J* = 8.0 Hz, 1H), 7.79 (t, *J* = 8.0 Hz, 1H), 7.51 (t, *J* = 8.0 Hz, 1H), 6.96 (dd, *J* = 8.0, 4.0 Hz, 1H), 6.89 (d, *J* = 4.0 Hz, 1H), 4.37–4.30 (m, 1H), 4.08–3.95 (m, 3H), 3.91–3.85 (q, *J* = 8.0 Hz, 1H), 2.18–2.09 (m, 1H), 2.06–1.94 (m, 2H), 1.85–1.76 (m, 1H); ^13^C NMR (100 MHz, CDCl_3_) *δ* = 161.55, 160.74, 152.49, 135.15, 134.88, 130.54, 127.76, 123.77, 121.11, 119.97, 112.90, 111.28, 102.27, 76.85, 70.86, 68.72, 28.19, 25.76; HRMS (ESI) *m*/*z* calcd for C_18_H_17_O_4_^+^[M + H]^+^: 297.1049; found: 297.1045. Chromatographic purity: 99.1% (HPLC).

3-((1,3-Dioxolan-2-yl)methoxy)-6H-benzo[c]chromen-6-one (**1o**): Using 2-(bromomethyl)-1,3-dioxolane as the starting material, the desired white solid **1o** was isolated 1.93 g, 69%). M.p. 135.6–137.8 °C. ^1^H NMR (400 MHz, CDCl_3_) *δ* = 8.35 (d, *J* = 8.0 Hz, 1H), 8.00 (d, *J* = 8.0 Hz, 1H), 7.94 (d, *J* = 8.0 Hz, 1H), 7.81–7.77 (m, 1H), 7.51 (t, *J* = 8.0 Hz, 1H), 6.96 (dd, *J* = 12.0, 4.0 Hz, 1H), 6.90 (d, *J* = 4.0 Hz, 1H), 5.34 (t, *J* = 4.0 Hz, 1H), 4.14–3.96 (m, 6H); ^13^C NMR (100 MHz, CDCl_3_) *δ* = 161.45, 160.29, 152.43, 135.02, 134.90, 130.54, 127.86, 123.84, 121.14, 120.01, 112.74, 111.61, 102.49, 101.66, 69.01, 65.41; HRMS (ESI) *m*/*z* calcd for C_17_H_15_O_5_^+^[M + H]^+^: 299.0841; found: 299.0839. Chromatographic purity: 98.6% (HPLC).

3-((3,5-Dimethoxybenzyl)oxy)-6H-benzo[c]chromen-6-one (**1p**): Using 1-(bromomethyl)-3,5-dimethoxybenzene as the starting material, the desired white solid **1p** was isolated (3.37 g, 99%). M.p. 133.8–135.0 °C. ^1^H NMR (300 MHz, DMSO-*d*_6_) *δ* = 8.23–8.11 (m, 3H), 7.82 (t, *J* = 9.0 Hz, 1H), 7.52 (t, *J* = 7.5 Hz, 1H), 7.00– 6.97 (m, 2H), 6.58 (d, *J =* 3.0 Hz, 2H), 6.40 (t, *J* = 3.0 Hz, 1H), 5.09 (s, 2H), 3.68 (s, 6H); ^13^C NMR (75 MHz, DMSO-*d*_6_) *δ* = 161.03, 160.94, 160.56, 152.43, 139.24, 135.76, 135.13, 130.13, 128.58, 125.22, 122.43, 119.73, 113.39, 111.34, 106.01, 102.87, 99.98, 70.04, 55.65; HRMS (ESI) *m*/*z* calcd for C_22_H_19_O_5_^+^[M + H]^+^: 363.1154; found: 363.1151. Chromatographic purity: 98.4% (HPLC).

3-((1,3-Dimethyl-1H-pyrazol-5-yl)methoxy)-6H-benzo[c]chromen-6-one (**1q**): Using 5-(bromomethyl)-1,3-dimethyl-1H-pyrazole as the starting material, the desired whitesolid **1q** was isolated (2.92 g, 97%). M.p. 165.1–167.6 °C. ^1^H NMR (300 MHz, CDCl_3_) *δ* = 8.34 (dd, *J* = 9.0, 1.5 Hz, 1H), 8.00–7.93 (m, 2H), 7.82–7.76 (m, 1H), 7.54–7.49 (m, 1H), 6.95 (m, 2H), 6.16 (s, 1H), 5.07 (s, 2H), 3.87 (s, 3H), 2.26 (s, 3H); ^13^C NMR (75 MHz, CDCl_3_) *δ* = 161.39, 159.70, 152.41, 147.46, 136.99, 134.98, 134.89, 130.57, 128.02, 124.00, 121.17, 120.01, 112.96, 111.84, 107.14, 102.50, 60.81, 36.53, 13.44; HRMS (ESI) *m*/*z* calcd for C_19_H_17_N_2_O_3_^+^[M + H]^+^: 321.1161; found: 321.1158. Chromatographic purity: 97.1% (HPLC).

3-(Pyridin-3-ylmethoxy)-6H-benzo[c]chromen-6-one (**1r**): Using 3-(bromomethyl)pyridine as the starting material, the desired yellow solid **1r** was isolated (1.42 g, 50%). M.p. 143.6–145.3 °C. ^1^H NMR (300 MHz, CDCl_3_) *δ* = 8.69 (d, *J* = 30.0 Hz, 2H), 8.35 (dd, *J* = 9.0, 3.0 Hz, 1H), 7.98 (dd, *J* = 12.0,6.0 Hz, 2H), 7.853–7.76 (m, 2H), 7.51 (t, *J* = 7.5 Hz, 1H), 7.37 (dd, *J* = 9.0, 3.0 Hz, 1H), 6.98 (dd, *J* = 9.0, 3.0 Hz, 1H), 6.97 (d, *J* = 3.0 Hz,1H), 5.15 (s, 2H); ^13^C NMR (75 MHz, CDCl_3_) *δ* = 161.39, 160.06, 152.50, 149.79, 149.07, 135.42, 134.95, 134.10, 131.74, 130.58, 127.97, 124.00, 123.65, 121.17, 120.03, 112.92, 111.75, 102.64, 67.94; HRMS (ESI) *m*/*z* calcd for C_19_H_14_NO_3_^+^[M + H]^+^: 304.0895; found: 304.0891. Chromatographic purity: 96.2% (HPLC).

3-(Cyclopentylmethoxy)-6H-benzo[c]chromen-6-one (**1s**): Using (bromomethyl)cyclopentane as the starting material, the desired yellow solid **1s** was isolated (2.13 g, 77%). M.p. 90.6–91.2 °C. ^1^H NMR (300 MHz, CDCl_3_) *δ* = 8.34 (dd, *J* = 9.0, 1.5 Hz, 1H), 7.98 (dd, *J* = 6.0, 1.2 Hz, 1H), 7.91 (d, *J* = 9.0 Hz, 1H), 7.79–7.74 (m, 1H), 7.51–7.45 (m, 1H), 6.90 (dd, *J* = 9.0, 3.0 Hz, 1H), 6.83 (d, *J* = 3.0 Hz, 1H), 3.89 (d, *J* = 6.0 Hz, 2H), 2.47 (m, 1H), 1.96–1.79 (m, 2H), 1.752–1.55 (m, 4H), 1.44–1.32 (m, 2H); ^13^C NMR (75 MHz, CDCl_3_) *δ* = 161.61, 161.19, 152.56, 135.25, 134.85, 130.52, 127.61, 123.67, 121.05, 119.89, 112.87, 110.87, 102.12, 72.67, 38.90, 29.48, 25.45; HRMS (ESI) *m*/*z* calcd for C_19_H_19_O_3_^+^[M + H]^+^: 295.1256; found: 295.1251. Chromatographic purity: 98.4% (HPLC).

3-((4-[Hydroxymethyl]benzyl)oxy)-6H-benzo[c]chromen-6-one (**1t**): Using (4-(bromomethyl)phenyl)methanol as the starting material, the desired white solid **1t** was isolated (2.40 g, 77%). M.p. 147.0–149.2 °C. ^1^H NMR (300 MHz, DMSO-*d*_6_) *δ* = 8.33 (d, *J* = 9.0 Hz, 1H), 8.29–8.21 (m, 2H), 7.96–7.90 (m, 1H), 7.65–7.60 (m, 1H), 7.49 (d, *J* = 9.0 Hz, 2H), 7.40 (d, *J* = 9.0 Hz, 2H), 7.12–7.07 (m, 2H), 5.29 (t, *J* = 9.0 Hz, 1H), 5.24 (s, 2H), 4.55 (d, *J* = 6.0 Hz, 2H); ^13^C NMR (75 MHz, DMSO-*d*_6_) *δ* = 160.95, 160.68, 152.45, 142.94, 135.79, 135.17, 130.15, 128.58, 128.24, 127.02, 125.21, 122.44, 119.72, 113.45, 111.27, 102.87, 70.14, 63.10; HRMS (ESI) *m*/*z* calcd for C_21_H_17_O_4_^+^[M + H]^+^: 333.1049; found: 333.1044.Chromatographic purity: 97.2% (HPLC).

3-(2-Morpholinoethoxy)-6H-benzo[c]chromen-6-one (**1u**): Using 4-(2-bromoethyl)morpholine as the starting material, the desired yellow solid **1u** was isolated (1.22 g, 40%). M.p. 100.8–102.8 °C. ^1^H NMR (300 MHz, CDCl_3_) *δ* = 8.35 (dd, *J* = 9.0, 1.2 Hz, 1H), 7.96 (dd, *J* = 18.0, 6.0 Hz, 2H), 7.78 (m, 1H), 7.50 (t, *J* = 7.5 Hz, 1H), 6.94–6.90 (dd, *J* = 9.0, 3.0 Hz, 2H), 4.18 (t, *J* = 6.0 Hz, 2H), 3.76 (t, *J* = 7.5 Hz, 4H), 2.85 (t, *J* = 4.5 Hz, 2H), 2.61 (t, *J* = 4.5 Hz, 4H); ^13^C NMR (75 MHz, CDCl_3_) *δ* = 161.50, 160.55, 152.52, 135.09, 134.91, 130.55, 127.80, 123.80, 121.10, 119.96, 112.88, 111.29, 102.25, 66.91, 66.25, 57.43, 54.13; HRMS (ESI) *m*/*z* calcd for C_19_H_20_NO_4_^+^[M + H]^+^: 326.1314; found: 326.1310. Chromatographic purity: 99.2% (HPLC).

#### 3.1.3. General Procedure for the Synthesis of 3-Hydroxy-6*H*-benzo[*c*]chromen-6-one (**II**)

Using 2-bromo-5-methoxybenzoic acid (60 mmol, 13.79 g) acid as the starting material, the synthesis method was similar to that of compound **I**, the desired yellow solid **II** was isolated (6.82 g, 47.0%). M.p. 258.4–261.7 °C. ^1^H NMR (300 MHz, DMSO*-d_6_*) *δ* = 10.22 (s*, *1H), 8.21 (d, *J* = 9.0 Hz, 1H), 8.09 (d, *J* = 8.0 Hz, 1H), 7.60 (d, *J* = 3.0 Hz, 1H), 7.49 (dd, *J* = 9.0, 3.0 Hz, 1H), 6.83 (dd, *J* = 9.0, 3.0 Hz, 1H), 6.75 (d, *J* = 3.0 Hz, 1H), 3.89 (s, 3H); ^13^C NMR (75 MHz, DMSO-*d*_6_) *δ* = 160.97, 159.41, 158.94, 151.62, 128.97, 124.57, 124.36, 123.99, 120.48, 113.55, 111.27, 109.96, 103.30, 56.01; HRMS (ESI) *m*/*z* calcd for C_14_H_10_O_4_^+^[M+H]^+^: 242.0579; found: 242.0575. Chromatographic purity: 97.2% (HPLC).

#### 3.1.4. General Procedures for Synthesis of Compounds **2a**–**w**

The anhydrous DMF (30 mL) was added to a 250 mL round bottom flask, compound **II** (9.4 mmol, 2.28 g), anhydrous K_2_CO_3_ (12.2 mmol, 1.7 g) and halides (12.2 mmol) were added, and the temperature was controlled at 80~120 °C, the reaction was stirred for 24 h. The reaction was monitored by TLC (PE:EA = 3:1). When the reaction was over, the reaction solution was to the ice-water mixture to obtain brown crude solid. After suction filtration and drying, the brown crude solid was purified by column chromatography to obtain compounds **2a**–**w**.

3,8-Dimethoxy-6H-benzo[c]chromen-6-one (**2a**): Using iodomethane as the starting material, the desired white solid **2a** was isolated (1.23 g, 51%). M.p. 146.2–148.8 °C. ^1^H NMR (400 MHz, DMSO-*d*_6_) *δ* = 8.22 (d, *J* = 9.0 Hz, 1H), 8.14 (d, *J* = 9.0 Hz, 1H), 7.58 (d, *J* = 3.0 Hz, 1H), 7.47 (dd, *J* = 9.0, 3.0 Hz, 1H), 6.96 (s, 1H), 6.94 (t, *J* = 4.0 Hz, 1H), 3.89 (s, 3H), 3.84 (s, 3H); ^13^C NMR (100 MHz, DMSO-*d*_6_) *δ* = 160.85, 160.83, 159.23, 151.55, 128.55, 12.50, 124.34, 124.31, 120.79, 112.69, 111.30, 111.21, 101.79, 56.15, 56.04; HRMS (ESI) *m*/*z* calcd for C_15_H_13_O_4_^+^[M + H]^+^: 257.0736; found: 257.0731. Chromatographic purity: 99.2% (HPLC).

3-Ethoxy-8-methoxy-6H-benzo[c]chromen-6-one (**2b**): Using bromoethane as the starting material, the desired white solid **2b** was isolated(1.14 g, 45%). M.p. 141.1–143.8 °C. ^1^H NMR (400 MHz, DMSO-*d*_6_) *δ* = 8.26 (d, *J* = 8.0 Hz, 1H), 8.17 (d, *J* = 8.0 Hz, 1H), 7.62 (d, *J* = 4.0 Hz, 1H), 7.51 (dd, *J* = 8.0, 4.0 Hz, 1H), 6.98 (s, 1H), 6.96 (t, *J* = 4.0 Hz, 1H), 4.13 (q, *J* = 8.0 Hz, 2H), 3.90 (s, 3H), 1.37 (t, *J* = 8.0 Hz, 3H); ^13^C NMR (75 MHz, DMSO-*d*_6_) *δ* = 160.89, 160.10, 159.23, 151.55, 128.61, 124.52, 124.39, 124.32, 120.78, 113.03, 111.32, 111.10, 102.20, 64.19, 56.05, 14.95; HRMS (ESI) *m*/*z* calcd for C_16_H_15_O_4_^+^[M + H]^+^: 271.0892; found: 271.0890. Chromatographic purity: 98.1% (HPLC).

8-Methoxy-3-propoxy-6H-benzo[c]chromen-6-one (**2c**): Using 1-bromopropane as the starting material, the desired white solid **2c** was isolated (1.39 g, 52%). M.p. 109.3–111.6 °C. ^1^H NMR (400 MHz, DMSO-*d*_6_) *δ* = 8.18 (d, *J* = 8.0 Hz, 1H), 8.09 (d, *J* = 8.0 Hz, 1H), 7.56 (d, *J* = 4.0 Hz, 1H), 7.45 (dd, *J* = 8.0, 4.0 Hz, 1H), 6.94–6.90 (m, 2H), 3.99 (t, *J* = 6.0 Hz, 2H), 3.88 (s, 3H), 1.75 (m, 2H), 1.00 (t, *J* = 8.0 Hz, 3H); ^13^C NMR (100 MHz, DMSO-*d*_6_) *δ* = 160.86, 160.23, 159.20, 151.52, 128.58, 124.46, 124.33, 124.27, 120.75, 113.00, 111.29, 111.08, 102.20, 69.95, 56.02, 22.37, 10.81; HRMS (ESI) *m*/*z* calcd for C_17_H_17_O_4_^+^[M + H]^+^: 285.1049; found: 285.1045. Chromatographic purity: 99.3% (HPLC).

3-(Sec-butoxy)-8-methoxy-6H-benzo[c]chromen-6-one (**2d**): Using 2-bromobutane as the starting material, the desired yellow solid **2d** was isolated (0.95 g, 34%). M.p. 64.5–66.7 °C. ^1^H NMR (400 MHz, DMSO-*d_6_*) *δ* = 8.21 (d, *J* = 8.0 Hz, 1H), 8.12 (d, *J* = 8.0 Hz, 1H), 7.59 (d, *J* = 4.0 Hz, 1H), 7.47 (dd, *J* = 8.0, 4.0 Hz, 1H), 6.95 (s, 1H), 6.92 (d, *J* = 4.0 Hz, 1H), 4.56–4.49 (m, 1H), 3.89 (s, 3H), 1.73–1.57 (m, 2H), 1.27 (d, *J* = 6.0 Hz, 3H), 0.94 (t, *J* = 8.0 Hz, 3H); ^13^C NMR (100 MHz, DMSO-*d_6_*) *δ* = 160.89, 159.40, 159.20, 151.62, 128.62, 124.57, 124.35, 124.27, 120.77, 113.84, 111.32, 111.01, 103.23, 75.18, 56.03, 28.90, 19.37, 9.94; HRMS (ESI) *m*/*z* calcd for C_18_H_19_O_4_^+^[M + H]^+^: 299.1205; found: 299.1201. Chromatographic purity: 98.7% (HPLC).

3-Butoxy-8-methoxy-6H-benzo[c]chromen-6-one (**2e**): Using 1-bromobutane as the starting material, the desired white solid **2e** was isolated (1.82 g, 65%). M.p. 102.5–104.4 °C. ^1^H NMR (300 MHz, DMSO-*d*_6_) *δ* = 8.26 (d, *J* = 9.0 Hz, 1H), 8.19–8.15 (m, 1H), 7.62 (d, *J* = 3.0 Hz, 1H), 7.51 (dd, *J* = 9.0, 3.0 Hz, 1H), 6.98 (s, 1H), 6.96 (t, *J* = 4.5 Hz, 1H), 4.07 (t, *J* = 6.0 Hz, 2H), 3.90 (s, 3H), 1.78–1.68 (m, 2H), 1.52–1.3840 (m, 2H), 0.95 (t, *J* = 7.5 Hz, 3H); ^13^C NMR (75 MHz, DMSO-*d*_6_) *δ* = 160.84, 160.25, 159.20, 151.52, 128.58, 124.43, 124.29, 124.24, 120.75, 112.99, 111.31, 111.08, 102.21, 68.21, 56.02, 31.06, 19.17, 14.14; HRMS (ESI) *m*/*z* calcd for C_18_H_19_O_4_^+^[M + H]^+^: 299.1205; found: 299.1201. Chromatographic purity: 99.1% (HPLC).

8-Methoxy-3-(pentyloxy)-6H-benzo[c]chromen-6-one (**2f**): Using 1-bromopentane as the starting material, the desired white solid **2f** was isolated (1.34 g, 46%). M.p. 73.1–75.5 °C. ^1^H NMR (300 MHz, DMSO-*d*_6_) *δ* = 8.23 (d, *J* = 9.0 Hz, 1H), 8.15–8.12 (m, 1H), 7.59 (d, *J* = 3.0 Hz, 1H), 7.49 (dd, *J* = 8.0, 3.0 Hz, 1H), 6.97–6.93 (m, 2H), 4.03 (t, *J* = 7.5 Hz, 2H), 3.89 (s, 3H), 1.78–1.69 (m, 2H), 1.44–1.34 (m, 4H), 0.93–0.88 (m, 3H); ^13^C NMR (75 MHz, DMSO-*d*_6_) *δ* = 160.89, 160.26, 159.22, 151.54, 128.62, 124.50, 124.39, 124.32, 120.77, 113.05, 111.29, 111.09, 102.21, 68.49, 56.04, 28.70, 28.13, 22.37, 14.40; HRMS (ESI) *m*/*z* calcd for C_19_H_21_O_4_^+^[M + H]^+^: 313.1362; found: 313.1359. Chromatographic purity: 98.5% (HPLC).

3-Isopropoxy-8-methoxy-6H-benzo[c]chromen-6-one (**2g**): Using 2-bromopropane as the starting material, the desired white solid **2g** was isolated (0.97 g, 36%). M.p. 88.3–90.7 °C. ^1^H NMR (300 MHz, DMSO-*d*_6_) *δ* = 8.21 (d, *J* = 9.0 Hz, 1H), 8.14–8.11 (m, 1H), 7.58 (d, *J* = 3.0 Hz, 1H), 7.48 (dd, *J* = 9.0, 3.0 Hz, 1H), 6.95–6.91 (m, 2H), 4.75 (m, 1H), 3.89 (s, 3H), 1.32 (d, *J* = 6.0 Hz, 6H); ^13^C NMR (75 MHz, DMSO-*d*_6_) *δ* = 160.89, 159.19, 159.05, 151.60, 128.60, 124.55, 124.34, 124.26, 120.76, 113.81, 111.30, 110.98, 103.20, 70.33, 56.03, 22.14; HRMS (ESI) *m*/*z* calcd for C_17_H_17_O_4_^+^[M + H]^+^: 285.1049; found: 285.1045. Chromatographic purity: 98.6% (HPLC).

3-(2-Hydroxyethoxy)-8-methoxy-6H-benzo[c]chromen-6-one (**2h**): Using 2-bromoethanol as the starting material, the desired yellow solid **2h** was isolated (1.51 g, 56%). M.p. 166.8–168.0 °C. ^1^H NMR (300 MHz, DMSO-*d*_6_) *δ* = 8.21 (d, *J* = 9.0 Hz, 1H), 8.15–8.11 (m, 1H), 7.57 (d, *J* = 3.0 Hz, 1H), 7.47 (dd, *J* = 9.0, 3.0 Hz, 1H), 6.98–6.94 (m, 2H), 4.98 (t, *J* = 6.0 Hz, 1H), 4.08 (dd, *J* = 6.0, 3.0 Hz, 2H), 3.89 (s, 3H), 3.77 (q, *J* = 6.0 Hz, 2H); ^13^C NMR (75 MHz, DMSO-*d*_6_) *δ* = 160.86, 160.25, 159.19, 151.50, 128.55, 124.48, 124.33, 124.26, 120.76, 113.05, 111.26, 111.14, 102.28, 70.56, 59.89, 56.02; HRMS (ESI) *m*/*z* calcd for C_16_H_15_O_5_^+^[M + H]^+^: 287.0841; found: 287.0837. Chromatographic purity: 98.8% (HPLC).

8-Methoxy-3-(2-(methylthio)ethoxy)-6H-benzo[c]chromen-6-one (**2i**): Using (2-bromoethyl)(methyl)sulfane as the starting material, the desired white solid **2i** was isolated (1.18 g, 40%). M.p. 110.1–111.8 °C. ^1^H NMR (300 MHz, DMSO-*d_6_*) *δ* = 8.27 (d, *J* = 9 Hz, 1H), 8.19 (d, *J* = 9.0 Hz, 1H), 7.62 (d, *J* = 3.0 Hz, 1H), 7.52 (dd, *J* = 9.0, 3.0 Hz, 1H), 7.03–6.98 (m, 2H), 4.26 (t, *J* = 6.0 Hz, 2H), 3.90 (s, 3H), 2.89 (t, *J* = 7.5 Hz, 2H), 2.18 (s, 3H); ^13^C NMR (75 MHz, CDCl_3_) *δ* = 165.60, 164.53, 164.05, 156.28, 133.29, 129.33, 129.13, 125.61, 117.84, 116.16, 116.12, 107.21, 72.70, 60.82, 37.28, 20.45; HRMS (ESI) *m*/*z* calcd for C_17_H_17_O_4_S^+^[M + H]^+^: 317.0769; found: 317.0765. Chromatographic purity: 97.8% (HPLC).

Ethyl 2-((8-methoxy-6-oxo-6H-benzo[c]chromen-3-yl)oxy)acetate (**2j**): Using ethyl 2-bromoacetate as the starting material, the desired white solid **2j** was isolated (1.58 g, 51%). M.p. 151.5–152.8 °C. ^1^H NMR (300 MHz, DMSO-*d_6_*) *δ* = 8.28 (d, *J* = 9.0 Hz, 1H), 8.21 (t, *J* = 6.0 Hz, 1H), 7.62 (d, *J* = 3.0 Hz, 1H), 7.52 (dd, *J* = 9.0, 3.0 Hz, 1H), 7.03– 6.99 (m, 2H), 4.92 (s, 2H), 4.20 (q, *J* = 6.0 Hz, 2H), 3.90 (s, 3H), 1.23 (t, *J* = 7.5 Hz, 3H); ^13^C NMR (75 MHz, DMSO-*d_6_*) *δ* = 168.85, 160.80, 159.42, 159.11, 151.39, 128.39, 124.63, 124.48, 124.40, 120.98, 113.01, 111.93, 111.42, 102.75, 65.41, 61.25, 56.09, 14.52; HRMS (ESI) *m*/*z* calcd for C18H17O6^+^[M + H]^+^: 329.0947; found: 329.0943. Chromatographic purity: 97.4% (HPLC).

3-(Cyclopentylmethoxy)-8-methoxy-6H-benzo[c]chromen-6-one (**2k**): Using (bromomethyl)cyclopentane as the starting material, the desired white solid **2k** was isolated (0.90 g, 30%). M.p. 146.1–148.2 °C. ^1^H NMR (300 MHz, DMSO*-d_6_*) *δ* = 8.26 (d, *J* = 9.0 Hz, 1H), 8.17 (t, *J* = 4.5 Hz, 1H), 7.62 (d, *J* = 3.0 Hz, 1H), 7.51 (dd, *J* = 9.0, 3.0 Hz, 1H), 6.99–6.95 (m, 2H), 3.94 (d, *J* = 6.0 Hz, 2H), 3.90 (s, 3H), 2.38–2.28 (m, 1H), 1.82–1.74 (m, 2H), 1.65–1.51 (m, 4H), 1.38–1.27 (m, 2H); ^13^C NMR (75 MHz, DMSO*-d_6_*) *δ* = 160.92, 160.44, 159.28, 151.59, 128.66, 124.55, 124.44, 124.39, 120.83, 113.15, 111.39, 111.15, 102.35, 72.61, 56.09, 38.88, 29.44, 25.41; HRMS (ESI) *m*/*z* calcd for C_20_H_21_O_4_^+^[M + H]^+^: 325.1362; found: 325.1360. Chromatographic purity: 98.1% (HPLC).

8-Methoxy-3-((tetrahydrofuran-2-yl)methoxy)-6H-benzo[c]chromen-6-one (**2l**): Using 2-(bromomethyl)tetrahydrofuran as the starting material, the desired white solid **2l** was isolated (1.71 g, 56%). M.p. 113.1–115.5 °C. ^1^H NMR (300 MHz, DMSO*-d_6_*) *δ* = 8.27 (d, *J* = 9.0 Hz, 1H), 8.18 (dd, *J* = 9.0, 3.0 Hz, 1H), 7.62 (d, *J* = 3.0 Hz, 1H), 7.51 (dd, *J* = 9.0, 3.0 Hz, 1H), 7.03–6.94 (m, 2H), 4.23–4.15 (m, 1H), 4.11–3.98 (m, 2H), 3.90 (s, 3H), 3.84–3.77 (m, 1H), 3.73–3.66 (m, 1H), 2.08–1.99 (m, 1H), 1.94–1.77 (m, 2H), 1.72–1.65 (m, 1H); ^13^C NMR (75 MHz, DMSO*-d_6_*) *δ* = 160.86, 160.14, 159.27, 151.51, 128.57, 124.52, 124.36, 120.83, 113.09, 111.35, 111.29, 102.39, 76.81, 71.08, 67.97, 56.06, 28.06, 25.67; HRMS (ESI) *m*/*z* calcd for C_19_H_19_O_5_^+^[M + H]^+^: 327.1154; found: 327.1150. Chromatographic purity: 99.2% (HPLC).

3-((1,3-Dioxolan-2-yl)methoxy)-8-methoxy-6H-benzo[c]chromen-6-one (**2m**): Using 2-(bromomethyl)-1,3-dioxolane as the starting material, the desired white solid **2m** was isolated (0.93 g, 30%). M.p. 147.9–150.1 °C. ^1^H NMR (300 MHz, DMSO-*d*_6_) *δ* = 8.28 (d, *J* = 9.0 Hz, 1H), 8.19 (d, *J* = 9.0 Hz, 1H), 7.62 (d, *J* = 3.0 Hz, 1H), 7.52 (dd, *J* = 9.0, 3.0 Hz, 1H), 7.04–6.99 (m, 2H), 5.24 (t, *J* = 3.0 Hz, 1H), 4.12 (d, *J* = 6.0 Hz, 2H), 4.01–3.96 (m, 2H), 3.90 (s, 3H), 3.89– 3.85 (m, 2H); ^13^C NMR (75 MHz, DMSO-*d*_6_) *δ* = 160.87, 159.76, 159.38, 151.49, 128.52, 124.63, 124.48, 124.45, 120.94, 113.12, 111.62, 111.41, 102.62, 101.57, 69.02, 65.03, 56.10; HRMS (ESI) *m*/*z* calcd for C_18_H_17_O_6_^+^[M + H]^+^: 329.0947; found:329.0943. Chromatographic purity: 97.6% (HPLC).

3-(Cyclohexylmethoxy)-8-methoxy-6H-benzo[c]chromen-6-one (**2n**): Using (bromomethyl)cyclohexane as the starting material, the desired white solid **2n** was isolated (1.85 g, 38%). M.p. 139.8–140.1 °C. ^1^H NMR (300 MHz, DMSO-*d*_6_) *δ* = 8.25 (d, *J* = 9.0 Hz, 1H), 8.18–8.14 (m, 1H), 7.61 (d, *J* = 3.0 Hz, 1H), 7.50 (dd, *J* = 9.0, 3.0 Hz, 1H), 6.99–6.95 (m, 2H), 3.90 (s, 3H), 3.87 (d, *J* = 6.3 Hz, 2H), 1.84–1.65 (m, 6H), 1.33–1.04 (m, 5H); ^13^C NMR (75 MHz, DMSO-*d*_6_) *δ* = 160.91, 160.43, 159.26, 151.58, 128.66, 124.54, 124.42, 124.37, 120.81, 113.10, 111.37, 111.12, 102.33, 73.67, 56.08, 37.43, 29.65, 26.49, 25.70; HRMS (ESI) *m*/*z* calcd for C_21_H_23_O_4_^+^[M + H]^+^: 339.1518; found: 339.1514. Chromatographic purity: 97.5% (HPLC).

3-(2-(1,3-Dioxolan-2-yl)ethoxy)-8-methoxy-6H-benzo[c]chromen-6-one (**2o**): Using 2-(2-bromoethyl)-1,3-dioxolane as the starting material, the desired white solid **2o** was isolated (1.22 g, 38%). M.p. 151.6–153.2 °C. ^1^H NMR (300 MHz, DMSO-*d*_6_) *δ* = 8.27 (d, *J* = 9.0 Hz, 1H), 8.20–8.17 (m, 1H), 7.62 (d, *J* = 3.0 Hz, 1H), 7.51 (dd, *J* = 9.0, 3.0 Hz, 1H), 7.00–6.96 (m, 2H), 5.02 (t, *J* = 4.5 Hz, 1H), 4.18 (t, *J* = 6.0 Hz, 2H), 3.95–3.91 (m, 2H), 3.90 (s, 3H), 3.83–3.79 (m, 2H), 2.07 (m, 2H); ^13^C NMR (75 MHz, CDCl_3_) *δ* = 161.64, 159.88, 159.13, 151.57, 128.66, 124.48, 123.11, 122.82, 120.96, 112.70, 111.33, 110.92, 102.20, 101.86, 65.02, 64.08, 55.76, 33.59, 29.73; HRMS (ESI) *m*/*z* calcd for C_19_H_19_O_6_^+^[M + H]^+^: 343.1103; found: 343.1101. Chromatographic purity: 96.9% (HPLC).

8-Methoxy-3-((tetrahydro-2H-pyran-4-yl)methoxy)-6H-benzo[c]chromen-6-one (**2p**): Using 4-(bromomethyl)tetrahydro-2*H*-pyran as the starting material, the desired yellow solid **2p** was isolated (1.79 g, 36%). M.p. 168.1–168.5 °C. ^1^H NMR (400 MHz, CDCl_3_) *δ* = 7.94 (d, *J* = 8.0 Hz, 1H), 7.87 (d, *J* = 8.0 Hz, 1H), 7.78 (d, *J* = 4.0 Hz, 1H), 7.39 (dd, *J* = 8.0, 4.0 Hz, 1H), 6.91 (dd, *J* = 8.0, 4.0 Hz, 1H), 6.86 (d, *J* = 4.0 Hz, 1H), 4.06 (dd, *J* = 12.0, 4.0 Hz, 2H), 3.94 (s, 3H), 3.88 (d, *J* = 8.0 Hz, 2H), 3.51–3.45 (m, 2H), 2.19–2.0 (m, 1H), 1.82–1.78 (m, 2H), 1.56–1.46 (m, 2H); ^13^C NMR (75 MHz, DMSO-*d*_6_) *δ* = 160.91, 160.29, 159.31, 151.59, 128.64, 124.60, 124.45, 124.41, 120.86, 113.14, 111.41, 111.25, 102.42, 72.99, 67.06, 56.10, 34.79, 29.62; HRMS (ESI) *m*/*z* calcd for C_20_H_21_O_5_^+^[M + H]^+^: 341.1311; found: 341.1307. Chromatographic purity: 97.4% (HPLC).

3-(Benzyloxy)-8-methoxy-6H-benzo[c]chromen-6-one (**2q**): Using (bromomethyl)benzene as the starting material, the desired white solid **2q** was isolated (1.04 g, 33%). M.p. 141.7–142.5 °C. ^1^H NMR (300 MHz, DMSO*-d_6_*) *δ* = 8.27 (d, *J = 9.0 Hz*, 1H), 8.20 (d, *J* = 9.0 Hz, 1H), 7.62 (d, *J* = 3.0 Hz, 1H), 7.51–7.48 (m, 3H), 7.45–7.33 (m, 3H), 7.10–7.04 (m, 2H), 5.22 (s, 2H), 3.90 (s, 3H); ^13^C NMR (75 MHz, DMSO*-d_6_*) *δ* = 162.53, 159.77, 158.99, 151.43, 136.98, 128.96, 128.33, 124.30, 121.58, 120.60, 113.25, 111.25, 102.70, 70.15, 55.98; HRMS (ESI) *m*/*z* calcd for C_21_H_17_O_4_^+^[M + H]^+^: 333.1049; found: 333.1045. Chromatographic purity: 98.7% (HPLC).

3-((4-[Hydroxymethyl]benzyl)oxy)-8-methoxy-6H-benzo[c]chromen-6-one (**2r**): Using (4-(bromomethyl)phenyl)methanol as the starting material, the desired white solid **2r** was isolated (1.26 g, 37%). M.p. 192.4–194.6 °C. ^1^H NMR (300 MHz, DMSO-*d*_6_) *δ* = 8.27 (d, *J* = 9.0 Hz, 1H), 8.19 (d, *J* = 9.0 Hz, 1H), 7.62 (d, *J* = 3.0 Hz, 1H), 7.51 (dd, *J* = 9.0, 3.0 Hz, 1H), 7.44 (dd, *J* = 6.0, 3.0 Hz, 2H), 7.35 (d, *J* = 8.0 Hz, 2H), 7.08–7.03 (m, 2H), 5.20 (t, *J* = 6.0 Hz, 3H), 4.51 (d, *J* = 3.0 Hz, 2H), 3.90 (s, 3H); ^13^C NMR (75 MHz, DMSO-*d*_6_) *δ* = 160.86, 159.87, 159.32, 151.49, 142.91, 135.27, 128.55, 128.18, 127.01, 124.57, 124.40, 120.88, 113.43, 111.44, 111.39, 102.84, 70.11, 63.11, 56.08; HRMS (ESI) *m*/*z* calcd for C_22_H_19_O_5_^+^[M + H]^+^: 363.1154; found: 363.1150. Chromatographic purity: 98.6% (HPLC).

3-((3,5-Dimethoxybenzyl)oxy)-8-methoxy-6H-benzo[c]chromen-6-one (**2s**): Using 1-(bromomethyl)-3,5-dimethoxybenzene as the starting material, the desired white solid **2s** was isolated (1.59 g, 43%). M.p. 153.6–155.3 °C. ^1^H NMR (300 MHz, CDCl_3_) *δ* = 7.89 (d, *J* = 9.0 Hz, 1H), 7.84 (d, *J* = 9.0 Hz, 1H), 7.73 (d, *J* = 3.0 Hz, 1H), 7.35 (dd, *J* = 9.0, 3.0 Hz, 1H), 6.96 (dd, *J* = 9.0, 3.0 Hz, 1H), 6.89 (d, *J* = 3.0 Hz, 1H), 6.59 (d, *J* = 3.0 Hz, 2H), 6.42 (t, *J* = 3.0 Hz, 1H), 5.05 (s, 2H), 3.91 (s, 3H), 3.80 (s, 6H); ^13^C NMR (75 MHz, DMSO-*d*_6_) *δ* = 161.04, 160.88, 160.82, 159.76, 159.33, 151.48, 139.36, 128.54, 124.62, 124.44, 120.90, 113.41, 111.51, 111.35, 106.65, 105.99, 102.85, 99.96, 70.00, 56.08, 55.67, 55.49; HRMS (ESI) *m*/*z* calcd for C_23_H_21_O_6_^+^[M + H]^+^: 393.1260; found: 393.1257. Chromatographic purity: 98.3% (HPLC).

3-((2-Fluorobenzyl)oxy)-8-methoxy-6H-benzo[c]chromen-6-one (**2t**):Using 1-(bromomethyl)-2-fluorobenzene as the starting material, the desired white solid **2t** was isolated (2.47 g, 75%). M.p. 203.6–204.9 °C. ^1^H NMR (300 MHz, DMSO-*d*_6_) *δ* = 8.30 (d, *J* = 9.0 Hz, 1H), 8.23 (d, *J* = 9.0 Hz, 1H), 7.64–7.58 (m, 2H), 7.52 (dd, *J* = 9.0, 3.0 Hz, 1H), 7.49–7.42 (m, 1H), 7.33–7.24 (m, 2H), 7.15 (d, *J* = 3.0 Hz, 1H), 7.07 (dd, *J* = 9.0, 3.0 Hz, 1H), 5.25 (s, 2H), 3.91 (s, 3H); ^13^C NMR (75 MHz, DMSO-*d*_6_) *δ* = 159.49, 159.27, 151.59, 130.17, 130.06, 129.80, 128.58, 124.55, 124.42, 124.37, 123.26, 122.89, 121.09, 115.70, 115.42, 112.90, 111.78, 111.00, 102.74, 64.17, 55.79; HRMS (ESI) *m*/*z* calcd for C_21_H_16_FO_4_^+^[M + H]^+^: 351.0954; found: 351.0950. Chromatographic purity: 97.6% (HPLC).

3-((1,3-Dimethyl-1H-pyrazol-5-yl)methoxy)-8-methoxy-6H-benzo[c]chromen-6-one (**2u**): Using 5-(bromomethyl)-1,3-dimethyl-1H-pyrazole as the starting material, the desired white solid **2u** was isolated (1.71 g, 52%). M.p. 184.6–186.6 °C. ^1^H NMR (300 MHz, DMSO-*d*_6_) *δ* = 8.29 (d, *J* = 9.0 Hz, 1H), 8.21 (d, *J* = 9.0 Hz, 1H), 7.63 (d, *J* = 3.0 Hz, 1H), 7.52 (dd, *J* = 9.0, 3.0 Hz, 1H), 7.15 (d, *J* = 3.0 Hz, 1H), 7.07 (dd, *J* = 9.0, 3.0 Hz, 1H), 6.19 (s, 1H), 5.23 (s, 2H), 3.90 (s, 3H), 3.76 (s, 3H), 2.12 (s, 3H); ^13^C NMR (75 MHz, CDCl_3_) *δ* = 165.59, 164.15, 164.09, 156.20, 150.86, 142.74, 133.24, 129.37, 129.23, 129.16, 125.71, 118.13, 116.53, 116.17, 111.87, 107.73, 65.66, 60.85, 18.37; HRMS (ESI) *m*/*z* calcd for C_21_H_19_N_2_O_4_^+^[M + H]^+^: 351.1267; found: 351.1263. Chromatographic purity: 98.7% (HPLC).

8-Methoxy-3-(pyrimidin-2-yloxy)-6H-benzo[c]chromen-6-one (**2v**): Using 2-bromopyrimidine as the starting material, the desired white solid **2v** was isolated (1.29 g, 23%). M.p. 212.3–214.0 °C. ^1^H NMR (300 MHz, DMSO-*d*_6_) *δ* = 8.69 (d, *J* = 6.0 Hz, 2H), 8.38 (dd, *J* = 9.0, 6.0 Hz, 2H), 7.68 (d, *J* = 3.0 Hz, 1H), 7.57 (dd, *J* = 9.0, 3.0 Hz, 1H), 7.38 (d, *J* = 3.0 Hz, 1H), 7.33 (t, *J* = 4.5 Hz, 1H), 7.27 (dd, *J* = 9.0, 3.0 Hz, 1H), 3.93 (s, 3H); ^13^C NMR (75 MHz, CDCl_3_) *δ* = 160.11, 158.86, 152.24, 149.99, 126.84, 123.51, 122.38, 122.22, 120.83, 117.40, 115.74, 114.80, 110.18, 109.98, 54.79; HRMS (ESI) *m*/*z* calcd for C_18_H_13_N_2_O_4_^+^[M + H]^+^: 321.0797; found: 321.0794. Chromatographic purity: 96.8% (HPLC).

3-(2-(1H-pyrazol-1-yl)ethoxy)-8-methoxy-6H-benzo[c]chromen-6-one (**2w**): Using 1-(2-bromoethyl)-1*H*-pyrazole as the starting material, the desired white solid **2w** was isolated (1.93 g, 61%). M.p. 159.0–161.1 °C. ^1^H NMR (300 MHz, DMSO-*d*_6_) *δ* = 8.32 (d, *J* = 9.0 Hz, 1H), 8.22 (d, *J* = 9.0 Hz, 1H), 7.87 (d, *J* = 3.0 Hz, 1H), 7.67 (d, *J* = 3.0 Hz, 1H), 7.58–7.53 (m, 2H), 7.05–6.98 (m, 2H), 6.32 (t, *J* = 3.0 Hz, 1H), 4.59 (t, *J* = 6.0 Hz, 2H), 4.50 (t, *J* = 4.5 Hz, 2H), 3.95 (s, 3H); ^13^C NMR (75 MHz, DMSO-*d*_6_) *δ* = 160.83, 159.59, 159.35, 151.49, 139.37, 131.06, 128.47, 124.61, 124.41, 120.90, 113.10, 111.60, 111.39, 105.70, 102.55, 67.55, 56.08, 50.95; HRMS (ESI) *m*/*z* calcd for C_19_H_17_N_2_O_4_^+^[M + H]^+^: 337.1110; found: 337.1106. Chromatographic purity: 98.9% (HPLC).

### 3.2. Enzymatic Assays

The in vitro inhibitory activity of compounds 1**a**–**u** and 2**a**–**w** against PDE2, which used Bio-cAMP as substrate, are listed in Table 1. The specific experimental method was reported below [32].

Preparation of 1*Rection Buffer: we took 1 mL 10*Rection Buffer, added 9 mL ultra-pure water to swirl and mix well, and put it on ice; preparation of PDE2 solution: 210 μL of 1*Rection Buffer were added into a centrifuge tube, then 30 μL PDE2 were added, mixed well, equal to 8-times dilution, and placed on ice; preparation of compound solution with gradient concentration: DMSO was used as the solvent to prepare 10 mM compound solution, and then diluted with 1*Rection Buffer. The solution with final concentrations of 40 nM, 20 nM, 10 nM, 5 nM, 2.5 nM, 1.25 nM, and 0.625 nM was prepared, respectively, and the solution was vortex mixed.

We took 2 μL of the diluted compound solution of each concentration into the microplate, added 4 μL PDE2 solution, numbered them A1–A9, added 2 μL 1*Rection Buffer and 4 μL PDE2 into A10, added 2 μL compound solution and 4 μL 1*Rection Buffer to A11, added 6 μL 1*Rection Buffer to A13, and ensured the three groups are parallel to each other. Centrifugation was carried out at 1000 R and 25 °C for about 2 min. The reaction time was 0.5 h in the microporous plate thermostatic oscillator. After the reaction, 4 μL Bio-cAMP was added to A1–A13, and centrifugation was carried out at 1000 R and 25 °C for about 2 min and reacted in a microporous plate thermoelectric oscillator for 1 h.

Anti-Camp Alohascreen Acceptor and Streptavidin Coated Donor Bead were purchased from Perkin Elmer Company according to response volume: 1* Immunoassay Buffer 549.32 μL and 3.70 μL anti-cAMP AlohaScreen Acceptor were collected under dark conditions. We took 1.98 μL Streptavidin coated Donor Bead, mixed well, added 15 μL mixture to each well after 1 h reaction, and reacted it in the dark for 1 h. After the reaction, we tested it with multilabel microplate detector. According to the detection results, the inhibition rate was calculated according to the dilution ratio of the experimental system and the formula, and the IC_50_ of compound was calculated by GraphPad Prism 5.

### 3.3. Cell Assays

#### 3.3.1. Solution Preparation

We added 943 µL of DMSO solution to 8.2 mg of dexamethasone acetate to obtain a 20 mM stock solution, froze it at −20 °C, and diluted it to 2 mM with serum-containing medium before use. The test compound was dissolved in DMSO to obtain a 20 mM stock solution and stored at −20 °C. Diluted with serum-containing medium to a concentration of 2 × 100, 2 × 50, 2 × 25, 2 × 12.5, 2 × 6.25, 2 × 3.125 μM before use. The control sample Bay 60-7550 stock solution with a concentration of 1 mM was diluted with serum-containing medium to a concentration of 2 × 0.1, 2 × 1, 2 × 2, and 2 × 4 μM before use.

#### 3.3.2. Cell Culture and Dexamethasone Intervention

After resuscitation, HT-22 cells were cultured in DMEM medium (containing 10% FBS) at 37 °C with 5% CO_2_. After the cells grew to more than 80% cell fusion, we passaged and seeded the plate, and the density of each well should be 10,000 cells/100 μL/well and cultured for 24 h. After the cells adhered to the wall, drug intervention was given.

#### 3.3.3. Drug Intervention

10 µL solution of 2 mM dexamethasone acetate was cultured at 37 °C for 30 min. 100 µL drug solution of 2 × 100, 2 × 50, 2 × 25, 2 × 12.5, 2 × 6.25, and 2 × 3.125 μM were added to make the final concentration of dexamethasone acetate 100 μM, and the final concentrations of the drug were 100, 50, 25, 12.5, 6.25, 3.125 μM. After preparation, it took another 24 h to continue the culture. There were 5 replicate wells for each sample, and the normal control group, dexamethasone acetate-affected group, and positive control group were set at the same time (final concentration 0.1, 1, 2, and 4 μM).

#### 3.3.4. MTT Color Experimental and Data Processing

After the incubation, 50 µL of MTT was added to each well, and it needed another 4 h to incubate. Discarded the upper matrix and the 150 μL DMSO was added. To dissolve the blue-violet precipitate completely, the shaker was used to shake for 10 min. Finally, the wavelength A 490 was measured. The data were expressed as mean ± sd (*n* = 5), and one-way anova analysis of variance was used for data statistics; *p* < 0.05 is defined as a significant difference.

## 4. Conclusions

In this paper, a series of new 6*H*-benzo[*c*] chromen-6-one derivatives were designed and synthesized with PDE2 as the potential therapeutic target, and the PDE2 inhibitory activity was tested at the enzyme level. The results showed that part of compounds exhibited good PDE2 inhibitory activity, such as 1f, 1h, 1i, 1l, 1s, 2e, and 2i. Among them, 1f has the highest PDE2 inhibitory activity with IC_50_ value of 3.67 ± 0.47 μM. The protective effects of compound 1**f** against corticosterone-induced HT-22 neurotoxicity were further verified. Compound 1**f** can protect HT-22 cells from corticosterone-induced death and rescue corticosterone-induced toxicity in a dose-dependent manner, which is probably associated with PDE2 inhibition. In the future, we will design and synthesize more active PDE2-inhibitors based on an analysis of the structure-activity relationship between compound 1**f** and 4HTX for the potential treatment of AD. In addition, the in vivo test would be performed in mouse models of AD to obtain more convincing and potentially therapeutic molecules.

## Figures and Tables

**Table 1:** In vitro PDE2 inhibitory activity of 6*H*-benzo[*c*]chromen-6-one derivatives 1 and 2.

Compound	PDE2 IC_50_ ^a^ (μM)	Compound	PDE2 IC_50_ ^a,b^ (μM)
**1a**	28.82 ± 7.8	**2a**	>50
**1b**	25.40 ± 5.13	**2b**	>50
**1c**	25.80 ± 5.45	**2c**	>50
**1d**	>50	**2d**	>50
**1e**	37.65 ± 4.9	**2e**	17.77 ± 4.07
**1f**	3.67 ± 0.47	**2f**	>50
**1g**	>50	**2g**	30.29 ± 7.38
**1h**	18.69 ± 2.72	**2h**	>50
**1i**	17.88 ± 1.87	**2i**	14.88 ± 1.78
**1j**	43.22 ± 3.67	**2j**	>50
**1k**	>50	**2k**	>50
**1l**	19.27 ± 2.38	**2l**	>50
**1m**	>50	**2m**	>50
**1n**	27.51 ± 1.49	**2n**	>50
**1o**	>50	**2o**	>50
**1p**	>50	**2p**	25.16 ± 6.91
**1q**	>50	**2q**	23.31 ± 4.28
**1r**	27.56 ± 2.14	**2r**	>50
**1s**	8.14 ± 0.54	**2s**	>50
**1t**	>50	**2t**	Insoluble
**1u**	>50	**2u**	29.39 ± 5.01
		**2v**	23.88 ± 545
		**2w**	>50

^a^ BAY 60-7550 was used as the reference compound with an IC_50_ of 8.4 nM. ^b^ Results are expressed as the mean of at least three experiments.

## Data Availability

Not applicable.
